# The relational institution: an ethnographic study of recovery orientation and relational engagement on a psychiatric rehabilitation ward in London

**DOI:** 10.1186/s12888-024-06140-0

**Published:** 2024-10-28

**Authors:** Henry J. Whittle, Ed Kiely, Isabel Millard, Sushrut Jadhav, Helen Killaspy

**Affiliations:** 1 Division of Psychiatry, University College London, London, UK; 2 Department of Anthropology, University College London, London, UK; 3 School of Geography, Queen Mary University of London, London, UK

**Keywords:** Rehabilitation, Recovery, Therapeutic relationships, Relational engagement, Psychosis, Schizophrenia, Audit, Demoralisation, Burnout, Racism

## Abstract

**Background:**

In the UK, inpatient psychiatric rehabilitation services for complex psychosis aim to provide recovery-orientated treatment to patients, with the goal of supporting sustained stepdown into community living. The extent to which rehabilitation services uphold this recovery orientation is associated with better outcomes. However, few studies have been able to ascertain what promotes or prevents recovery orientation in inpatient settings.

**Methods:**

We conducted an ethnographic study of treatment on a National Health Service (NHS) psychiatric rehabilitation ward in London over six months during August 2022-February 2023. Data were collected through participant observation and semi-structured interviews with 9 patients and 14 staff members. Fieldnotes and interview transcripts were analysed using situational analysis.

**Results:**

Our analysis highlights the importance of what we term ‘relational engagement’ between staff and patients to nurture and sustain recovery-orientated treatment. This relational engagement was embodied through small acts of genuine human connection grounded in mutual acceptance and affective bonding; close attention to detail that communicated curiosity and respect; and recognition, appreciation, and encouragement of the slow and gradual progress that characterises recovery in complex psychosis. Yet, this relational engagement was often limited or foreclosed by the social environment of the ward and the wider institutional context. Limiting elements included the dominance of hospital logics geared towards high-throughput acute treatment and risk management; the presence of audit culture that led to a level of standardisation curtailing more genuine human connection; and staff demoralisation driven by events on and off the ward, including system-wide crises and more localised conflicts and disturbances. Some of these conflicts involved discrimination, most prominently anti-Black racism and homophobia, reflecting wider structural inequalities that characterise inpatient psychiatric populations and the healthcare workforce.

**Conclusion:**

Relationships, often under-prioritised in mental health services, were a key cornerstone of recovery-orientated treatment on a psychiatric rehabilitation ward. The shaping of therapeutic relationships amounted to an active process of relational engagement, which may be afforded or constrained by complex social elements requiring careful consideration in inpatient psychiatry. These social elements go beyond more surface-level factors such as staff training, knowledge, or attitudes and may require structural and system-level interventions.

## Introduction

Approximately 20–25% of people diagnosed with a psychotic illness will go on to develop ‘complex psychosis’ [1]. This relatively new term, recently endorsed by the UK’s National Institute for Health and Care Excellence (NICE) [1], refers to the presence of schizophrenia, bipolar affective disorder, or a related psychotic diagnosis with treatment-resistant symptoms and impairments in daily functioning. These impairments arise from the combination of clinical features associated with this illness profile, including chronic positive symptoms (delusions, hallucinations, etc.), negative symptoms (avolition, social withdrawal, etc.), illness-related cognitive impairments (particularly executive dysfunction), physical and mental health comorbidities, neurodevelopmental conditions (such as autism or attention deficient hyperactivity disorder), medication side effects, and/or alcohol and substance use. Within the UK’s National Health Service (NHS), the recognition of this severely disabled population has facilitated a specialised focus on their treatment needs, which are increasingly delivered through psychiatric rehabilitation pathways for complex psychosis [1]. These clinical pathways recognise that for this population specifically, sustained community living may require a longer initial inpatient rehabilitation phase, to ensure that patients are at a stable enough point in their recovery to optimise the chance of a successful community discharge.

Inpatient rehabilitation for complex psychosis involves intensive multidisciplinary treatment over an average length of stay of 9–12 months and is associated with sustained discharges from hospital for the majority [2]. Treatment goals generally focus on optimising symptoms and then improving patients’ daily occupational functioning such as self-care, cooking, cleaning, budgeting, and socialising, in preparation for step-down to a more independent community setting (such as a community rehabilitation unit or supported accommodation). Much of this work is done through occupational therapy and personalised care by nurses and support workers to encourage improvements in self-care and engagement in structured activity, both within the hospital and in the local community [1].

Yet, despite this consensus around what rehabilitation for complex psychosis involves and its clinical effectiveness, few studies have been able to establish what the key therapeutic ingredients of rehabilitation services are. One factor known to be associated with good outcomes in rehabilitation is the recovery orientation of the service. The recovery concept was developed in the 1980s, through patient-led initiatives, to move treatment goals away from symptom reduction and towards the person’s own unique vision for living a meaningful life despite the impacts of the illness [3–5]. It was subsequently operationalised into an approach to service delivery [6], which is widely agreed to include the following: treatment negotiated between patients and clinicians; facilitating patients’ engagement in meaningful activity; empowering patients to promote their autonomy, dignity, and self-management; supporting patients to reclaim their identity, overcome stigma, and develop self-acceptance; and maintaining hope and optimism [7]. While concerns have been raised by critics, including some service users, that aspects of the recovery concept—particularly the emphasis on independence and autonomy—have been appropriated to expedite discharge and cut longer term provision in some mental health services [8–11], here we refer to its deployment in rehabilitation pathways designed specifically to ensure comprehensive, longitudinal support over a timeframe of years. In this context, the extent to which rehabilitation services operate with a recovery orientation has been found to be positively associated with sustained and stable community living for patients [12].

However, exactly *how* staff nurture and maintain this recovery orientation, and what factors afford or constrain it, remains unclear. The ideals of the recovery movement have proved difficult to institutionalise in many mental health systems [13], and changing or improving staff practice in inpatient rehabilitation services also appears to be challenging. For example, a randomised control trial of a staff training intervention across 40 inpatient rehabilitation units failed to improve patients’ engagement in meaningful activity, likely because staff reverted to their habitual way of doing things after the training ended [14]. This raises the question of what structural and institutional factors might be determining staff practice beyond simply their knowledge and training.

In this study, we sought to investigate the recovery orientation of an NHS inpatient rehabilitation unit using ethnographic methods. Ethnography is a qualitative method particularly suited to this research question because it attends to actual practice on the ground, in all its complexity, as opposed to interviews or focus groups where staff might report best practice rather than the reality of the situation.

## Theoretical framing

The theoretical grounding of research into inpatient psychiatric institutions dates back to the post-war period, most famously in Goffman’s ‘total institution’ [15]. Contrasting with Goffman’s treatise, more recent research has highlighted the ‘permeability’ of contemporary psychiatric units, where intermittent or sustained contact with the outside world via the local community is more common [16]. Both theories, however, depict the psychiatric institution as operating relatively autonomously and uniformly, in isolation from wider political, economic, cultural, and discursive trends. Another tradition of hospital ethnography, meanwhile, argues that hospitals are continuously shaped by the societies in which they are embedded [17]. This study sits broadly in that latter tradition. Here, the theoretical framing reflects the strong emphasis in contemporary social science on the *situatedness* of social phenomena: how knowledges are constructed within heterogenous networks of actors at particular times in particular places, embodied by people who both shape and are shaped by dominant social structures, discourses, and other nonhuman elements [18].

We draw on a range of contributions to this broad theoretical grounding. These include Foucauldian discourse formation and fields of practice, which convey how individual and collective subjects are constituted through disciplining in discursive regimes of knowledge/power, contingent on time and place. These discursive regimes, correspondingly, are sustained by their ongoing enactment in practice [19–21]. Also influential is the explicit importance of materialities in science and technology studies, where the human and nonhuman are recognised to co-constitute the social world, illuminating how nonhuman elements structurally condition social relations [22, 23]. The open-ended and dynamic interconnectedness of non-hierarchical assemblages is equally important, standing in opposition to the stable micro-/meso-/macro-level hierarchies characteristic of modernist theories [24, 25]. This broad theoretical framing is tied together through the “methods/theory package” of *situational analysis* (detailed below) [18], which aims to fracture open simplistic narratives and explore the messy complexity of social phenomena such as that under study here—the way in which treatment played out on a psychiatric rehabilitation ward in London.

## Methods

This was a clinical ethnographic study of Apollo Ward (pseudonym), an NHS psychiatric rehabilitation ward in London, conducted over six months between August 2022 and February 2023. Ethnography is an anthropological methodology that involves the primary researcher immersing themselves in their chosen anthropological field over time and forming longitudinal relationships with research participants. *Clinical* ethnography is purposefully oriented towards analysis with clinical as well as anthropological value [26].

### Research setting

Apollo Ward is a 16-bed mixed gender ward located within a larger inpatient psychiatric unit. It represents the first step of a longer NHS rehabilitation pathway extending into local community rehabilitation services. Patients meeting criteria for complex psychosis are usually transferred in from acute wards. As a rehabilitation ward, Apollo Ward is similar in setup to the acute wards that constitute the rest of the hospital but with additional facilities such as an occupational therapy kitchen, activities room, and games room.

### Recruitment

Any patient admitted to Apollo Ward and any staff member working there during the fieldwork period met the inclusion criteria. There were no exclusion criteria. The primary researcher (HJW, a psychiatrist trained in medical anthropology) approached all patients present on the ward at the start of fieldwork, enrolling 9 out of 16. The staff roster was large and changeable, so we recruited a core of regulars, looking for a range of seniority. The 14 staff enrolled included healthcare assistants, nurses, doctors, therapists, and managers. The managers did not all work frontline on the ward, such that we could “study up” the institution [27]. The primary researcher was already familiar with the hospital and ward logistics, as well as some patients and staff members, from previous clinical work in the hospital. During recruitment and throughout fieldwork he was therefore mindful of the possibilities of role confusion and patients feeling obliged to participate. To mitigate, all staff and patients were explicitly made aware and regularly reminded of the primary researcher’s professional identity as a psychiatrist and that he was not currently working as a doctor, but was conducting a research study in which participation was entirely voluntary with no consequences for not participating. No financial incentives were provided for participation because the project had no study budget (besides HJW’s salary). While this omission was structurally driven, we acknowledge the potentially problematic ethical implications of not compensating participants for their time [28].

### Data collection

The primary method of data collection was participant observation. During the six months of fieldwork, the primary researcher spent most days (including some weekends, evenings, and nights) in the ward’s communal areas, conversing with participants and observing everyday ward life. At times he accompanied patients into the local community (most were detained under the Mental Health Act but had leave approved for such excursions). He consistently endeavoured to carry out an *embodied* participant observation, attempting to pay conscious attention to his body/mind and his emotional reactions to events and participants [29–31]. These data were captured in contemporaneous jot notes alongside transcripts of conversations and descriptions of events, all of which were written down verbatim in a pocket notebook as quickly as possible after they had occurred. These jot notes were then written up into fieldnotes, usually that same day.

Participant observation was supplemented by semi-structured interviews with 21 of the 23 participants (the remaining two declined), mostly conducted at the end of fieldwork. For three patients who were discharged from Apollo Ward towards the end of fieldwork, the interview was conducted shortly after discharge. Semi-structured interviews were conducted wherever the participant felt comfortable and were audio-recorded and transcribed verbatim. Fieldnotes and transcripts were anonymised and uploaded to the qualitative data management software NVivo.

### Data analysis

The “methods/theory package” employed for data analysis is situational analysis. This is a recent elaboration on grounded theory that was developed in response to critiques of grounded theory [18]. Situational analysis proceeds through the making of various analytic maps, sketched and re-sketched iteratively during data collection. These maps capture the human actors, nonhuman actants, discursive constructions, and political, sociocultural, symbolic, spatial, and temporal elements present in the anthropological field, as well as the social worlds and positionalities observed. The primary researcher engaged this process throughout data collection to produce initial analyses. This iterative mapping of participant observation data was then triangulated through open coding of data from the semi-structured interviews [32], a third of which were double-coded by other members of the research team (EK, a health geographer and qualitative researcher with expertise in ethnography of mental health services; and IM, a rehabilitation psychiatrist with training in qualitative research methods). The final triangulated analysis was produced through discussion among the research team until consensus.

This analytic process was supplemented by reflexive analysis throughout [33]. Reflexive memos were made in a daily diary alongside jot notes throughout fieldwork and incorporated into the analytic process. The primary researcher’s awareness of his own professional identity was particularly significant, as it conveyed a degree of socialisation into the culture of NHS mental healthcare and the discourse of clinical psychiatry. This unavoidably colours the analysis with a clinical lens, which supports clinical applicability but may foreclose alternative perspectives. His identity as a White, male, financially stable researcher with no psychiatric diagnosis also meant that during moments in fieldwork when social inequalities were illuminated—e.g. psychiatric restriction, stigma, racism, patriarchy, or class hierarchy—he could rely only on his research training and not on experiential knowledge. The research plan and draft analyses were discussed with a group of mental health service users experienced in clinical research, which improved important aspects of the analysis.

### Ethics

This study was given NHS ethics approval in August 2022. Our ethics application included provisions to minimise perpetuating the systemic exclusion of people with complex psychosis from clinical research, which has led to their under-representation in the clinical evidence base [34, 35]. To this end, we were granted approval not to exclude patients automatically when their illness affected their capacity to consent to the research (e.g. due to cognitive impairments or severe negative symptoms). If such patients were freely and willingly engaging with data collection without any distress, they could still be included in the study contingent on an established NHS ethics process that involves a designated consultee providing informed written consultation. For all participants with capacity to consent, informed written consent was obtained. Regardless of participation, all patients on Apollo Ward were aware of the primary researcher’s role and no fieldnotes were written directly about patients who were not participating.

## Results

Table 1 shows the demographic and clinical characteristics of patient and staff participants.

**Table 1:** Demographic and clinical characteristics of participants

	Patients (*n* = 9)		Staff (*n* = 14)	
**Age**	**Average**	**Range**	**Average**	**Range**
*Years*	47	29–65	45	30–59
**Gender**	***n***	**%**	***n***	**%**
*Male*	5	56	5	36
*Female*	4	44	9	64
**Ethnicity**	***n***	**%**	***n***	**%**
*White British*	3	33	5	36
*White Other*	3	33	3	21
*Asian or Asian British*	2	22	1	7
*Black or Black British – African*	1	11	3	21
*Mixed Ethnicity*	0	0	2	14
**Primary Diagnosis**	***n***	**%**		
*Schizophrenia*	3	33		
*Schizoaffective Disorder*	6	67		
**Legal Status**	***n***	**%**		
*Section 3*	7	78		
*Informal*	2	22		
**Staff Group**			***n***	**%**
*Support*			4	29
*Nursing*			5	36
*Senior*			5	36
**Time Worked on Ward**			**Average**	**Range**
*Years*			4.7	0.25–18

To help protect the confidentiality of more specialist staff, we have grouped them into support workers (healthcare assistants, activity workers, and students); nurses (staff nurses and charge nurses); and senior staff (managers, doctors, and psychological and occupational therapists). Table 2 shows pseudonyms with gender and ethnicity by patient/staff group.

**Table 2:** Participant pseudonyms with gender and ethnicity by group

	Patients	Support Staff	Nursing Staff	Senior Staff
Pseudonyms	Alison Andrew Ayla Fiona Hamza Janet Jim Shepherd Tristan	India Karolina Mateo Vicky	Ana Imelda Isaac Samuel Tim	Angela Emma Magda Michael Sharon
Genders	5 male 4 female	1 male 3 female	3 male 2 female	1 male 4 female
Ethnicities	3 White British 3 White Other 2 Asian or Asian British 1 Black or Black British – African	2 White Other 1 Black or Black British – African 1 Mixed Ethnicity	1 White British 1 Asian or Asian British 2 Black or Black British – African 1 Mixed Ethnicity	4 White British 1 White Other

Patients on Apollo Ward generally had a significant level of functional impairment. Due to negative symptoms, many would spend their days in passive activity such as sleeping or watching television if left to choose. Many struggled with self-care. There was a wide range of ability when it came to shopping, preparing food, and cooking, but functional abilities in these domains were generally low. Motivation for engagement in activity groups and structured community integration was often very limited.

The majority of patients were detained for involuntary treatment under Sect. 3 of the Mental Health Act, meaning that they either did not agree to their hospitalisation or they lacked capacity to consent to it. While they had not been detained in the first instance by clinicians working on Apollo Ward, most patients did not perceive this to be an important distinction, and they understood that the ward’s consultant psychiatrist was now ultimately in charge of their treatment and discharge plans. The two participants who were informal patients had previously been detained but had subsequently agreed to stay on a voluntary basis, allowing them greater freedom to come and go from the hospital during the day. However, their engagement with staff did not differ radically from the rest of the sample, and they still had to abide by the ward’s general rules such as restrictions on potentially risky possessions, alcohol use, smoking, curfews, etc. This meant that their daily experiences were not starkly different either.

In general, despite their histories with involuntary detention, the patients did not actively object to the more medicalised interventions. For example, most accepted medications willingly—although few managed their own medication regimens, which were mostly left to the nursing staff to administer. What some patients disputed more frequently were the ward’s restrictions on their everyday movements and habits, for example their leave, personal possessions, or food choices. Still, this was highly variable: for some patients with severe negative symptoms like Alison, Hamza, and Jim, treatment involved supporting them to leave the hospital *more*, or to exercise greater agency in their daily routines.

Given that the concept of recovery assumes a degree of active input from patients, as well as broad alignment of treatment goals, this mix of passivity, avolition, and disagreement about treatment created myriad tensions and contradictions that had to be navigated carefully on the ward. Staff needed to find a way of balancing assertiveness with collaboration, structure and consistency with flexible person-centredness, and friendly familiarity with boundaried professionalism. Where staff did this effectively, it was overwhelmingly due to the skill and expertise with which they built and maintained therapeutic relationships with patients—what we term ‘relational engagement.’ This is demonstrated in the following quote from patient Tristan, reflecting on his time on Apollo Ward after he was stepped down to a community rehabilitation unit towards the end of fieldwork:

“Some of [the staff members] were just really nice. They tried to make you feel at home. […] I guess they gave people respect, which was good. Yeah, just, like, trying to get you involved in activities and stuff, like arts and crafts, exercise groups [*pause*] trying to get you out into the community. […] Like sometimes I feel like [*pause*] getting you medication is the main part of their job. But I think [*pause*] you know, the good ones try and encourage you in other areas [*pause*] which is, yeah, I think it makes you feel more positive.”

The centrality of staff in this quote captures the relational nature of recovery work on the ward. This relational engagement also stands out because most of the patients on Apollo Ward had few other social relationships: they had small social networks, few friends, and little engagement with family. The reasons for this appeared to be multifactorial, involving negative symptoms, stigma, and gradual social withdrawal driven by repeated psychiatric admissions—as Tristan himself put it, “it’s difficult to know where you stand with people.” On Christmas Day, for example, most patients remained on the ward and did not see family, despite the ward staff’s encouragement, either because it felt too stressful or they had lost contact. This social isolation, which presented further barriers to recovery-orientated treatment and community integration, stood in contrast to the best examples of relational engagement with staff. Our analysis here therefore focuses on (1) unpacking this relational engagement between staff and patients and (2) the structural/institutional relegation and de-prioritisation of this relational engagement on the ward, which ultimately meant that it was neither as prominent nor as consistent as it could have been.

### Relational engagement on Apollo Ward

There were three aspects of this relational engagement that stood out. The first aspect was a microscopic attention to detail that allowed treatment to be as flexible, accommodating, and individualised as possible. While this was modelled by care reviews in which the team paid close attention to every aspect of the patient’s care and treatment plan, it was most present in innumerable moments of fleeting patient-staff interactions on the ward, seemingly of little significance individually but which collectively scaffolded the personalisation essential to recovery-orientated treatment. Here is one example from HJW’s fieldnotes, involving a support worker, Mateo, and a patient, Shepherd, in a smoothie-making group:

Mateo gets Shepherd to cut some of the fresh fruit; he says they’ll do one each, Mateo a pear and Shepherd an orange. Shepherd struggles with this. I notice his hands are trembling (perhaps extrapyramidal side effects). He is trying to cut the peel off and Mateo tries to show him the best technique, but he can’t help but make a bit of a mess of it. Juice is flowing over the table and the cut isn’t smooth, the orange pulp looks like it’s been attacked. After a few minutes of struggle Mateo comes to his aid and takes the knife and orange off him, completing the task while carefully demonstrating how, like a parent might to a child. It’s a moment I don’t think much of, but one that Mateo returns to after Shepherd leaves the room.

“I made a mistake there,” says Mateo, “I gave him the hardest one. I should have given him the pear. Luckily he took it well. These are the details that nobody thinks of, that you have to think about. It’s like when someone loses to someone of low status, it can cause problems.”

I ask him to clarify that last point.

“It’s like Hamza [another patient],” he says. “Everyone thinks of him as low status. But when we play games, sometimes he’s good at them and he wins. And when someone who thinks they are higher status loses to him, it’s a problem.”

“Like it’s humiliating?” I ask.

“Exactly. If you know the FA Cup, it’s like what they call a ‘giant killing’.”

Although a small example, this extract underlines Mateo’s thoughtfulness about the implications of even the most mundane interactions for patients, as well as how everyday events unfolding on the ward might affect group dynamics, confidence, and future engagement with staff. It is only through such close attention to detail, facilitated through relationship-building, that recovery-orientated treatment could unfold.

This attention to detail could also only be facilitated by having consistent staff who were given the time and space to nurture these long-term relationships actively. Illustrating the consequences of *not* meeting these requirements is the following quote, where support worker India admonished temporary bank staff for misunderstanding the details of Janet’s chronic delusions regarding ‘Queen Victoria,’ a prominent persecutory figure in her psychotic experience who she perceived to be responsible for almost all the unpleasant things that had happened in her life:

“Sometimes you’ll have bank staff come up and be like, ‘How are you, Queen Victoria?’ And it’s like, do you not understand how much she hates her?! You can’t call her Queen Victoria!”

Ethnographically, this attention to detail was not only important for thinking through treatment plans but communicated a level of respect and curiosity upon which deeper relations could be built.

A second aspect of relational engagement was an appreciation of the slow, barely perceptible progress that characterises psychiatric rehabilitation, which needs adequate time to unfold. As one staff member put it, “you have to remember about mental illness, the steps are so small and gradual that sometimes you almost can’t see them.” To be consolidated and sustained, this progress had to be noticed and appreciated by staff, and it then had to be encouraged and reinforced. Even the primary researcher, with the time and space to observe closely, often found his conscious awareness of the patients’ recoveries diminished in the daily bustle of ward life. Many staff members, however, were adept at this.

For example, one patient, Alison, had not showered, changed her clothes, or spoken to anyone for many months prior to admission. When she took her first shower, several months after moving onto Apollo Ward, HJW wrote in his fieldnotes that the staff celebrated it “as if they’ve just won a competition.” In her interview, support worker Karolina reflected on Alison slowly becoming more involved in changing her bedding:

“Like, Alison, now she’s coming to me and helping me to do the bed. And before [*trails off then laughs*]. You know, it’s slowly, slowly, slowly. And you need to involve them to—‘Oh, come’—if she’s just doing the pillow case, I said ‘Oh well done, you do good!’ And, you know, she’s happy, she starts to be happy, because ‘Oh, I did this.’”

Similarly, Mateo’s appraisal is captured in the following fieldnote:

We head up to the park, where Alison sits in silence, expressionless, on the bench. At some point Mateo leans into me and comments to me how much of a shift it is that Alison is even out here. “At the start, she wouldn’t even talk to anyone. Now, she’s having showers, she’s here. Incredible.”

A senior staff member’s reaction to Alison’s first attendance at a care review in person, nearly six months into her admission, was similar. The following fieldnote captures an exchange about 30 s into the review:

“Why are you doing this?” Alison asks him. “You’re a student.”

“I’m the doctor,” Michael replies gently.

“No you’re not, you’re a student,” mutters Alison, and abruptly stands up and walks straight out the door again.

Everyone looks at Michael, who smiles and shrugs. He’s very happy with the progress—she came into the review, even if it was for about 30 s. Those are the small gains.

Finally, a key ingredient of good relational engagement was a notable degree of affective bonding and mutual acceptance with patients. Several patients described staff members as “friends,” perhaps the only friends some of them had, and certain staff were highly skilled at treading the line carefully between maintaining professional boundaries and providing an affective warmth, curiosity, and reciprocity akin to friendship or even familial relations. Again, small, fleeting moments from HJW’s fieldnotes bring these relations to life, as in the tender father-son-like relationship of Mateo and Hamza, a severely disabled patient with prominent negative symptoms and hebephrenic features:

I sit with Hamza a while as he eats his chicken and chips.

Mateo pops into the dining area: “Don’t tell him all your secrets, Hamza,” he jokes. “Keep some for yourself.” Mateo looks at me. “Did he tell you about his dancing? He must have danced for about 1 hour. You know the song ‘500 miles’? ‘*And I would walk 500 more!’* [*singing*]. This was on.” He points at the takeaway. “He deserves it today.”

Mateo leaves the dining room. I look at Hamza and he looks back at me and grins. He starts laughing. I laugh back and start singing the song, and he raises his hands above his head while he sits, moving them in time with the music.

The sisterly camaraderie of support worker India with patients Fiona and Janet, who she took out to a café for Fiona’s birthday, is another example:

We see Fiona, Janet, India, and Fiona’s befriender on the way down to the caff for Fiona’s birthday.

“’Eyy, happy birthday!” we chorus.

“We’re having a girly trip,” says India, smiling. “Hey, Jan! Wait for us!” she calls after Janet, who is off down the hill.

The combination of these ingredients of attention to detail, appreciation for slow progress, and genuine human connection created an environment frequently described by patients as calmer, more friendly, more welcoming, and more homely than acute psychiatric wards—which one patient described as “dark, tense, hard.” Crucially, the quality of these relationships, built up over time, also allowed staff to push patients more assertively towards greater independence and community integration without unduly irritating, frustrating, or upsetting them. When we went bowling in the local community, for example, Hamza was reluctant to go, before ultimately enjoying and gaining much from the experience:

Hamza takes some persuading. Initially a yes, he changes his mind when it gets closer to the time. The staff members each try to re-convince him in turn; Angela tries; even Magda tries. But in the end it is Imelda who gets there, by telling him that he doesn’t actually have to bowl. He can just sit there if he wants. So he relents and nods to her increasingly more assertive questions about whether he’ll go.

Similarly, here is senior staff member Angela’s report on patient Jim, prior to a care review:

“I think he has made improvements since he’s been on the ward. He does respond well to structured activity, for example the walking group, getting out and about. Now we want to focus on discharge and community activities.” She explains how she tried to raise the possibility of him going to a MIND group but he really shut her down. “I left it with him that I wasn’t going to give up,” she says with a grin.

These examples, both of which ultimately supported community integration in line with recovery-orientated treatment, would have been impossible without Imelda or Angela’s established relations with these patients, and their intimate knowledge of what the patients’ barriers might be and how far they could push them towards activity and independence. Accordingly, this relational engagement directly facilitated the recovery orientation.

### The relegation of relational engagement

Yet, many staff members, in their interviews, described how they were unable to embody this relational engagement nearly as much or as well as they would have liked. This raised the important question of why they could not. In our analysis, three mechanisms stood out.

First, the dominant structures and discourses governing the psychiatric hospital (and wider mental health services) seemed to clash with those underpinning this relational engagement (and psychiatric rehabilitation in general). These dominant discourses and structures, which set the services up to support high-throughput acute treatment, conveyed that economically, hospitalisation is expensive; culturally, lengthy inpatient treatment is anachronistic; and clinically, risk management is the priority. There were many ethnographic examples attesting to these priorities, which clashed with the slower, more flexible, person-centred requirements of rehabilitation:

Magda [senior staff member] says that when she first arrived on the ward [from working on an acute ward], all she could think of was the cost to the hospital—a hangover from the dominant framings animating the acute wards: “This person is costing X hundred pounds a night for the bed, why are we just keeping them here? How much of a waste is this!” She says she’s come around from that way of thinking now, and adopted more of a ‘rehab mentality’, but it’s taken some time.

Nurse Isaac captured the difference: “On the acute ward, [the patients] are being prevented to do some things. On a rehab ward, [they] are *allowed*.” Many staff members’ training, however, had inculcated more comfort with the ‘preventing’ than the ‘allowing’. Nurse Tim described how during his early days on Apollo Ward he had had to reconsider the dominant logics he had been socialised into:

“There was certain things that I kind of thought, ‘God, should that be allowed?!’ […] I mean it’s good that she’s able to do that [knitting], but at the same time I just thought, that’s a *risk*.”

This speaks to the ‘default’ clinical framings prioritised by the wider institution, also seemingly reflected in the trust’s investment:

Magda calls this “the forgotten ward.” She explains how when she first arrived, the ward didn’t—and still doesn’t—have any of the benefits of investment that was provided to the acute wards. She gives the examples of big interactive patient flow screens in the nursing offices and individual laptops for staff. These were things that were given out to the acute wards, but not here. This ward, by contrast, is using “old-fashioned” white boards and old, slow, desktop IT systems.

The clashes between these dominant structures/discourses and those of rehabilitation had two important effects. First, relational engagement was effectively relegated to a quasi-optional ‘extra’, to be done when the more ‘core’ tasks of inpatient mental healthcare—documentation, risk assessments, observations, medication rounds, responding to other wards, escorting on leave, safety huddles, etc.—had been completed. As nurse Tim put it:

“A lot of the time [is spent] doing your documentation, doing your notes, then updating your care plans and your risk assessments. […] Having a one-to-one [with a patient] sometimes seems to be a difficult time. Because if I take a patient and sit [with them] […] then someone will want to know where I am or what I’m doing. […] And I really want to sit here and have this conversation, but then you feel like you can’t because something else happens that maybe is more of a priority.”

The second effect is that having to navigate the tensions created by this clash inevitably led to inconsistencies between staff members—e.g. one staff member might turn a blind eye to a patient coming back late from leave in the community if they felt their experience had been beneficial for independence and community integration, whereas another staff member might enforce the boundary more firmly. This was often experienced as confusing for patients and staff alike and sowed discord, which was usually resolved by retreating to and re-emphasising the boundary (to the detriment of a more flexible, person-centred approach).

Another important mechanism undermining relational engagement was the prominence of ‘audit culture’ on the ward, where *doing* and *showing* tended to be held in higher regard than *relating* and *reflecting.* At morning meetings, for example, the primary researcher was often struck by the importance given to discreet tasks that needed completing that day—as senior staff member Sharon put it, “feeling like it has to be kind of task-orientated, so, you know, there are *things* that need to be *done* on the wards.” While this is perhaps unavoidable to a degree, there were prominent ethnographic examples of how the bureaucratisation and standardisation inherent to this approach undermined the relational aspects of rehabilitation. Senior staff member Angela, for example, lamented that when she had started working on Apollo, her manager had told her the most important tasks were the standardised assessments of functioning done at admission and discharge to evidence improvement, which should constitute the bulk of her work. When it came to engaging with patients between those assessments, however—that is, the therapeutic work that would actually lead to the improvement—she was instructed to delegate to someone more junior.

To illustrate the extent to which this standardisation can sap the genuine human curiosity from patient-staff interactions, consider the following fieldnote regarding the standardised form to establish the ‘patient view’ carried out before care reviews:

Imelda comes over and asks to go through the patient sheet with Andrew, ahead of his care review. Andrew barely engages. She persists, but he keeps losing attention, and it’s clearly a pretty meaningless exercise for him. In the end they arrive at the following:

**How are you feeling?**

“Like shit.”

**How are you getting on with other people?**

“Like a cunt.” (When prompted ‘with staff?’ specifically: ) “Acceptable. They raped me.”

**Are your physical needs being met?**

“Yeah alright.”

**Are plans going ok for your social needs?**

“Yeah alright.”

**What about treatment?**

“I just want to take it with me.”

**Are there enough activities each day to make good use of time without overwhelming you?**

“I don’t want to.”

**What are your (the patient’s) own views and plans?**

“I don’t have any, I’ll wait for them to sort it out for me.”

**Has a carer’s assessment been offered or completed?**

“Yeah.”

None of these answers provide much clinically useful information, nor do they authentically reflect who Andrew is or what his perspectives were at that time. Yet, if the emphasis had been on Imelda to find out this information more organically, she likely could have done so in a manner more empowering for Andrew.

Another notable example was the weekly community meeting, held ostensibly to ensure that patients had an opportunity to voice their opinions about life on the ward and contribute meaningfully to its programmes. The hospital hierarchy, who considered this important material for regulatory inspections, had mandated that it be done in a rigid format for audit purposes. Senior staff member Emma explained:

“We *have* to have community meetings every week, and they *have* to be minuted, and they *have* to be actioned, and when people don’t do that, it looks like we’re very uncaring, and we’ll get marked down for that, it looks like we’re not following what people want. […] So, when you try to say to staff, you know, ‘Can you do this because it does make an impact?’ I think, I *understand*, they think it looks very tick-boxy, you know?”

Meanwhile, this was nurse Imelda’s opinion on the community meeting:

“It’s one of the saddest things I’ve ever seen. […] It’s so *tired*. But like, if you’d hold a community meeting, I think, like [*pause*] what do *they* [the patients] want to talk about? Like most of it, really, the running theme for me, is it’s more of what *we* want to talk about, it’s more of what *we* want to do, it’s more of what *we* think is best for them. As opposed to what *their* concerns are, what *they’re* passionate about. […] They’re not interested! Because we don’t talk about things they’re interested in! So they don’t want to participate!”

A final mechanism undermining relational engagement, and perhaps the most prominent during fieldwork, was the demoralisation of ward staff. Much of this was shaped by the post-pandemic context of the wider NHS, with fieldnotes reflecting the ongoing “nursing strikes, paramedic strikes, and upcoming ballots for junior and senior doctors. The overall feeling is that people are fed up, short-staffed, and underappreciated.” However, more proximally, demoralisation arose due to feeling undervalued and having little opportunity to process or reflect on the challenges of working with complex psychosis. During fieldwork, HJW asked nurse Imelda whether the staff had any space to process their emotions:

“Absolutely zero,” she says.

I remark that this is maybe why I get the impression some people feel burnt out.

“Err, yep!” she says, laughing at how obvious that is.

Again, there were wellbeing checklists during ward meetings every morning, but their standardisation undermined any sense of genuine care for staff, as captured in HJW’s fieldnote:

At the end of the meeting, the staff go through their usual checklist. “Is everyone feeling safe?” “Yes,” they chant in unison. “Does everyone know where the ligature points are?” “Yes,” etc. It strikes me, in the light of all that’s happened recently, how meaningless these routines become if they’re repeated *ad nauseum*. Multiple staff have told me how they often haven’t felt safe—and yet every morning they’re asked if they feel safe, and I’ve never heard anyone say anything other than, “Yes.”

The most junior staff, who often did the most relational engagement, also faced all the challenges inherent to this work: the sustained effort it requires to support people with severe avolition to engage in activities, for example, or the emotional fallout of managing low-level irritability that erupts into violence from time to time. They felt poorly recognised and remunerated for the thought they put into their work and consequently relied on more intrinsic motivations to sustain relational engagement. A sense of demoralisation was often bubbling beneath the surface, however, as captured in this quote from Mateo in reference to the NHS trust hierarchy:

“They may think Band 3 is not intelligent, is not educated [*pause*] the way they email or order us or treat us. But we are able to see things. And we are able to judge. And we are able to see injustices.”

The experience of India, who at recruitment had spoken very fondly of her job, demonstrates the precariousness of this situation. The following fieldnote was taken around Christmas time:

India doesn’t seem herself. She seems dazed and frantic at the same time, like an image of helplessness—patients in the corridor keep shouting her name because they want things, and it’s like she has to be in several places at the same time. She doesn’t seem herself, like she’s on the edge of giving up altogether. […] Ten minutes later and she comes back in the room, looking like she’s been crying. […] “I’m burnt out. I’m so burnt out. I’m working so much. They ask for so much, all of them [patients and other staff]. And I’m just overworked. All these long days, and my coursework, and then I get home, and guess what? I’m a single mum [*starts crying*]. There’s no one to wash *my* fucking pants, do you know what I mean? I feel like I’m gonna quit. I can’t do it anymore.”

This demoralisation also intersected with wider structural inequalities, eroding motivation to engage even further for those affected. From time to time, the irritability and occasional violence on the ward was inflamed with discriminatory language or targeted towards staff members with particular protected characteristics. During the fieldwork, the most prominent examples in this regard involved anti-Black racism, and there were also several homophobic incidents. Sometimes these aggressions were primarily driven by psychosis and sometimes they were not, but they were always inevitably complicated by the patients’ own feelings of powerlessness around admission and restriction. This complexity is captured in the following quote, which one of the patients said to HJW:

“The staff here never listen to you, the Black nurses. They’re horrible people, the Africans—do nothing but hold you down and inject you. It takes two days training to be a support worker, it’s a joke.”

Without much reflective space to process them, these incidents stoked tensions on the ward, contributed to divisions between groups of staff members (some of whom felt unsupported by others), and undermined people’s motivation in their work. For some Black staff, this was further aggravated by the moral injury of having to restrain what they perceived to be a disproportionate number of Black patients in the wider hospital when on duty. Nurse Ana described the consequences for relational engagement:

“I’ve seen it loads, discrimination against staff and patients, it’s the same. And it causes harm. Like for me, there’s the physical harm, but also I believe it causes real *psychological* harm. Like, if you’re not gonna look after *my* mental health, then that’s why some people are just gonna come to work, do their job, go. They’re not gonna talk to the patients.”

Sharon, one of the senior staff members, described how she thought this erosion of relational engagement ended up playing out on the ward in psychological terms:

“Once you’re in threat mode, you know, it’s hard to be broadly thoughtful about people, because once that system’s going, the whole of your attentional focus is very narrow. And it’s all that, you know, ‘better safe than sorry,’ quick decisions, you know, make your mind up very quickly. So I think it gets a bit circular really, because I think sometimes that can lead to [staff] responses that put or keep the people [patients] who are on the ward in that mode as well, and that can lead to all sorts of interactions that might be less helpful.”

Collectively, then, swimming against more dominant structures and discourses in the hospital, audit culture, and staff demoralisation seemed to undermine the relational engagement so thoughtfully curated at other times. This was not lost on the patients. Fiona remarked to HJW mid-conversation one day, “see, you stop me and talk to me. If you can do it, I don’t know why none of the other staff can?” Similarly, Janet remarked, after seeing a particular staff member:

“She’s too professional, too emotionally detached. She doesn’t give anything to her patients! Lots of them are in this place. There has to be some basic human contact! They’re too professional and too frigid.”

And clinically, when these therapeutic relations were significantly undermined, recovery-orientated treatment on Apollo Ward proved difficult. “I feel like I do these assessments and find out exactly what [the patients’] interests are and what activities they need,” senior staff member Angela told HJW, “and then [*shrugs*] nothing happens.”

## Discussion

This ethnographic study of a psychiatric rehabilitation ward in London illuminates the skilled relational engagement of staff with patients as a key therapeutic ingredient of recovery orientation, which is one of the few known predictors of good outcomes in rehabilitation services [12]. Relational engagement here was an active but under-acknowledged labour, embodied through small acts by predominantly junior staff, mainly in such peripheral spaces as corridors, kitchens, or on leave from the ward. It was characterised by a close attention to detail that facilitated curious, respectful, and person-centred treatment; by an appreciation and active sustaining of the slow, gradual progress seen in rehabilitation; and by a mutual acceptance and affective bonding grounded in genuine human connection. Yet, at the same time, this relational engagement was often limited or foreclosed by aspects of the social environment of the ward and the wider institutional context. These included a predominant institutional orientation set up to optimise high-throughput acute treatment and risk management; the inadvertent depersonalising effects of audit culture and regulatory governance; and the demoralisation of staff. The latter arose through a combination of difficult political-economic context, structural inequality, and fallout from the under-acknowledged challenges of working with complex psychosis.

These findings anchor the rehabilitation ward (and, by extrapolation, the wider psychiatric institution) as a saliently *relational* institution, in a dual sense—first, in underlining the centrality of interpersonal relations as a key therapeutic ingredient; and second, in highlighting how relations between diverse social elements, arising both inside and outside the hospital walls, collectively condition the institution’s functioning.

### A relational institution

The importance of clinician-patient relationships in psychiatry has been known for over a century [36]. The therapeutic alliance, which was first described by Freud [37] and later elaborated by Rogers [38], predicts psychotherapy outcomes robustly [39]. It is understood in Bordin’s pantheoretical model [40] as comprising *shared goals*, *shared tasks*, and an *affective bond* between patient and clinician [41]. Affective bonding emerged in our study as a key component of relational engagement, grounded in curiosity, respect, and connection. This evokes previous research on the importance of emotional attunement, epistemic humility, and relationality for cultivating clinical empathy with patients [42, 43]. Meanwhile, the other two components highlighted—attention to detail and appreciation of slow progress—perhaps capture the relational work required to generate shared goals and tasks in the challenging clinical context of complex psychosis, where negative symptoms, cognitive impairments, stigma, involuntary detention, disagreements about treatment, and previous traumatic experiences with mental health services all play a potentially obstructive role. That such strong therapeutic relationships were possible in this challenging milieu speaks to the relational expertise of the Apollo Ward staff. It also resonates with Mol’s seminal analysis of care, in which she argues that good healthcare does not depend on maximising patient choice and autonomy but entails a persistent, collaborative tinkering to attune complex treatments to complex lives [44]. While this will always be challenging in the context of inpatient psychiatry, it was made possible on Apollo Ward by the relational engagement we have described, through which staff could overcome the apparent paradox between involuntary treatment and the recovery orientation. This allowed them to carve out mutually acceptable paths forward, even in the face of fundamental disagreements about the need for ongoing hospital admission.

The clinical implications of this finding are bolstered by studies beyond psychiatric rehabilitation. Empirical health services research shows consistently that the therapeutic relationship is a reliable predictor of outcomes in psychiatry [45], including for severe mental illness and psychosis [46, 47]. In a systematic review of patient experiences of inpatient mental health services internationally, the most consistently reported theme concerned relationships with staff [48]. Similarly, a user-led qualitative study of people previously admitted to NHS psychiatric hospitals in London highlighted relationships as the primary determinant of participants’ inpatient experiences [49]. Yet, despite this vast historical and contemporary evidence base, relationships remain underemphasised and deprioritised within mental health services [50]. The effects of this are felt unevenly. Compared to other psychiatric inpatient and outpatient groups, for example, longer-term hospitalised patients with schizophrenia rated the quality of their therapeutic relationships lowest [51].

Previous studies have highlighted important barriers to therapeutic relationships from the perspective of inpatients, including poor communication, feeling unsafe, experiencing coercion, limited cultural competency, and unhelpful or inconsistent staff attitudes [48, 49]. Yet, this literature has been appraised as lacking contextual information about wider systems to help explain what determines the quality of therapeutic relationships [48]. One effect of this omission is to leave the impression that frontline staff have greater agency to change inpatient mental health services than they perhaps do, leading to over-emphasis on staff training interventions with little thought about what structural, institutional, discursive, and wider situational elements might constrain relational engagement. Our study, in contrast, describes a complex assemblage (that is, an apparent coming-together of heterogenous elements in time and space [52]) on Apollo Ward, which may be relevant to other inpatient psychiatric settings given the elements involved.

### A contemporary psychiatric assemblage

The interrelated social elements that limited relational engagement in our study were observed on Apollo Ward during 2022–2023 but reach beyond its walls and stretch further back in time. The dominant logics governing inpatient mental healthcare, for example, are rooted in deinstitutionalisation, the ideal of community care, and the symbol of the asylum as emblematic of the dark ages of psychiatry [10]. These discourses help sustain an institutional preference for shorter admissions and a scepticism towards lengthier hospitalisations such as those of inpatient rehabilitation [10]. Closely related is the salience of risk management, which emerged alongside the closure of the asylums and the transition to community care [53]. With this change came a transformation in the professional subjectivity of clinicians, who were now liable to be held individually accountable for the identification, evaluation, and reduction of risk to and from their patients [53, 54]. Similarly, the vast proliferation of formal monitoring, reporting, and quality assurance processes in public and private institutions in the UK and elsewhere since the 1980s, including the NHS, has been well documented [55]. One unhelpful effect of this phenomenon is that organisations are liable to become increasingly focused on the metrics by which they are assessed (e.g. the minutes of the Apollo Ward community meeting) rather than more qualitatively or professionally judged outcomes (e.g. Imelda’s perception of the community meeting) [56]. Importantly, in highlighting these elements, which are engrained into the political and economic structures of the UK [57], we are not necessarily denouncing or undermining their individual purposes. Rather, our study demonstrates how dominant they are in practice, how they may inadvertently undermine therapeutic relationships, and how they may collectively clash with the time, space, patience, collaboration, and flexibility required of recovery-orientated rehabilitation.

Another possible effect of these phenomena is contributing to disparities between what professional staff value in their work and what they are recognised and rewarded for. On Apollo Ward this contributed to staff demoralisation, which emerged as a key barrier to relational engagement and recovery-orientated treatment. Mental healthcare staff appear at high risk of stress and burnout anyway due to the inherent challenges of the work, particularly when treating patients who are involuntarily detained [58]. In our study, this was also exacerbated by the political and economic context of fieldwork, including post-pandemic fatigue, high inflation, and a pervasive perception of NHS crisis, underfunding, and staff shortages, alongside unprecedented industrial action. Yet, there were other contributing factors. Both inpatient psychiatric treatment and the NHS workforce are characterised by structural inequalities, particularly related to race and ethnicity. Patients from certain minoritised ethnic groups, particularly those of African and African-Caribbean heritage, are disproportionately subject to involuntary detention and entering services via the criminal justice system [59]. The NHS, in turn, is sustained by staff from minoritised ethnic groups—many of whom have also migrated from lower- and middle-income countries—who are more likely to report bullying, abuse, or harassment from patients and colleagues and are under-represented in senior positions (as in our sample) [60]. Yet, there is often little acknowledgement of the racialised relations this situation creates, both in terms of structural racism embedded into the power hierarchies of services and the interpersonal racism and microaggressions that can manifest bidirectionally between staff and patients (or indeed within those groups) [61].

On Apollo Ward, many more junior or frontline staff perceived that space to reflect on, process, or discuss racism and other forms of discriminatory abuse—or indeed *any* emotional or interpersonal challenges that arose from working with complex psychosis—was *de facto* deprioritised. The patients’ main avenues for discussing such issues (e.g. the community meeting or ‘patient view’ form) were similarly limited. Omissions like these might easily trigger cascades of interrelated challenges for staff and patients alike, such as demoralisation, burnout, mutual loss of trust, conflicts and inconsistencies within teams, increasing use of coercion, and the disintegration of therapeutic relationships and recovery-orientated treatment. Such scenarios echo and update the lessons of older psychoanalytically informed studies that described how ‘collective disturbances’ were liable to spread around the wards of asylums, leading to intermittent breakdowns of morale and institutional functioning [62].

Importantly, similar challenges might arise in many healthcare settings for severe mental illness, which underlines the clinical relevance of our findings beyond inpatient rehabilitation. At the same time, the sustained and intensive staff-patient contact of Apollo Ward seemed to amplify the pressure collectively exerted by these social elements, which was liable to escalate rapidly without the natural breaks of community treatment or the release valve of imminent discharge from acute wards. Yet, crucially, this sustained contact is also unique to inpatient rehabilitation, facilitating a degree of relational engagement rarely possible elsewhere in mental health services. We are arguing that this unique aspect of inpatient rehabilitation deserves careful attention to ensure that recovery-orientated treatment can flourish in settings like Apollo Ward.

## Strengths and limitations

The strengths of our study lie in its ethnographic methodology. The primary researcher is dual trained as a psychiatrist and anthropologist. While this likely foreclosed some avenues of data collection and analysis, it allowed him to navigate the clinically complex environment of Apollo Ward and collect rich, clinically applicable data from participants with complex psychosis, a group under-represented in clinical and qualitative research. Our use of situational analysis also allowed us to engage with the full social complexity of a rehabilitation ward, analyse recovery-orientated treatment in real time, and theorise the multitude of interrelated elements shaping relational engagement.

Limitations also come with this methodology. This was a single ward in London, meaning lessons for the wider practice of psychiatric rehabilitation must be extrapolated and then applied to different settings. Similarly, only nine patients were recruited into the study, meaning we may have missed important alternative viewpoints. For example, our patient sample, although representative of the ward’s demographics during fieldwork, was predominantly White, meaning that data on racism may have been skewed away from discrimination towards patients, which certainly occurs and is an important barrier to therapeutic relationships [61]. This should prompt us to wonder who is excluded even from services such as Apollo Ward—that is, whose voices are missing still?

## Conclusion

In inpatient rehabilitation services for complex psychosis, the length of admission is longer than on acute wards, the level of psychopathology is severe, and patients have often had challenging experiences with mental health services. Where staff are provided with the space, time, resources, and support to cultivate rich therapeutic relationships, recovery-orientated treatment may flourish. Conversely, dominant logics of inpatient psychiatry, audit culture, and staff demoralisation may constrain this relational engagement and recovery orientation. Supporting psychiatric rehabilitation services in this context is likely to be complex, but an accurate understanding of the issues at play is the first step. Intervention may then be required at multiple policy and service levels, both locally and nationally. Among other aims, useful initiatives might help to carve out institutional niches where rehabilitation logics can be protected; empower staff to put therapeutic relationships at the heart of clinical practice; and prioritise collective and localised conversations, acknowledgements, and reflections around the conflicts and social inequalities that erode relational engagement and ultimately undermine recovery-orientated treatment.

## Data Availability

The datasets generated and/or analysed during the current study are not publicly available. This is due to the risks of compromising the confidentiality of participants given how detailed the qualitative data are.
